# Self-Assembled Bifunctional Copper Hydroxide/Gold-Ordered Nanoarray Composites for Fast, Sensitive, and Recyclable SERS Detection of Hazardous Benzene Vapors

**DOI:** 10.3390/nano13132016

**Published:** 2023-07-06

**Authors:** Yanyan Lu, Xuzhou Yuan, Cuiping Jia, Biao Lei, Hongwen Zhang, Zhipeng Zhao, Shuyi Zhu, Qian Zhao, Weiping Cai

**Affiliations:** 1 Key Laboratory of Materials Physics, Anhui Key Laboratory of Nanomaterials and Nanotechnology, Institute of Solid State Physics, HFIPS, Chinese Academy of Sciences, Hefei 230031, China; sa21168128@mail.ustc.edu.cn (Y.L.); lbiao@issp.ac.cn (B.L.); zpzhao@issp.ac.cn (Z.Z.); zhushuyi@issp.ac.cn (S.Z.); zhaoqian@issp.ac.cn (Q.Z.); wpcai@issp.ac.cn (W.C.); 2 Science Island Branch of Graduate School, University of Science and Technology of China, Hefei 230026, China; 3 Shandong Hengcheng Testing Technology Co., Ltd., Yantai 261400, China; 4 School of of Economics and Management (SEM), Weifang University of Science and Technology, Weifang 262700, China; jiacuiping2023@126.com

**Keywords:** VOCs, bifunctional copper hydroxide/gold ordered nanoarray composites, self-assembly, fast and sensitive SERS detection, recyclable SERS substrate

## Abstract

Volatile organic compounds (VOCs), particularly monoaromatic hydrocarbon compounds (MACHs), pose a potential risk to the atmospheric environment and human health. Therefore, the progressive development of efficient detection methodologies is a pertinent need, which is still a challenge at present. In this study, we present a rapid and sensitive method to detect trace amounts of MACHs using a bifunctional SERS composite substrate. We prepared an Au/SiO_2_ enhanced layer and a porous Cu(OH)_2_ adsorption layer via microfluidic-assisted gas-liquid interface self-assembly. The composite substrate effectively monitored changes in benzaldehyde using time-varying SERS spectra, and track-specifically identified various VOCs such as benzene, xylene, styrene, and nitrobenzene. In general, the substrate exhibited a rapid response time of 20 s to gaseous benzaldehyde, with a minimum detection concentration of less than 500 ppt. Further experimental assessments revealed an optimum Cu(OH)_2_ thickness of the surrounding adsorption layer of 150 nm, which can achieve an efficient SERS response to MACHs. Furthermore, the recoverable and reusable property of the composite substrate highlights its practicality. This study presents a straightforward and efficient approach for detecting trace gaseous VOCs using SERS, with significant implications in the designing of SERS substrates for detecting other VOCs.

## 1. Introduction

Volatile organic compounds (VOCs) are widely recognized for their detrimental impact on human health and the environment [1,2]. Consequently, prominent environmental safety institutions such as NIOSH, EPA, and EU-OSHA have formulated guidelines to limit human exposure to VOCs in indoor and occupational settings [3,4,5]. Specific attention should be given to monoaromatic hydrocarbons (MACHs), especially benzene, toluene, benzaldehyde, and styrene, due to their high toxicity, ability to infiltrate living areas, and potential for immediate and long-term health risks even at extremely low concentrations (sub ppb) [6,7]. Therefore, developing accurate, rapid, portable, and real-time monitoring methods for trace amounts of MACHs is crucial in protecting human health. Despite the high sensitivity and accuracy of conventional detection methods for VOCs, such as gas chromatography (GC) [8], gas chromatography-mass spectrometry (GC-MS) [9,10] and fluorescence analysis [11], they are frequently expensive, complex, and time-consuming. Additionally, several portable and innovative technologies developed in recent years, including semiconductor gas sensors [12], electrochemical gas sensors [13], and optical gas sensors [14] are widely used for rapid detection of VOCs. However, their performance in terms of recognition and sensitivity is often unsatisfactory. Thus, there remains a demand for VOCs detection methods that are highly efficient, portable, and accurate to fulfill the expectations.

Surface-enhanced Raman spectroscopy (SERS) has found widespread use in numerous fields including analysis [15], sensing [16,17,18], and catalysis [19] due to its rapid response, high sensitivity, ability to recognize fingerprints, and portable detection [20,21,22]. SERS is expected to enable fast and efficient trace detection of VOCs due to the resonance coupling between the incident laser with a certain excitation wavelength and the precious metal substrate (such as Au, Ag, or Cu) that forms a powerful local electromagnetic field, resulting in several orders of magnitude amplification of Raman signals of the molecules in this region [21]. Currently developed precious metal SERS substrates with different nanostructures have shown a strong LSPR enhancement, including periodic nanostructures, porous structures, etc. [16,18,23]. However, most VOCs molecules have small optical scattering cross-sections and weak Raman signals, and they interact weakly with plasmonic metals, which makes it difficult for them to be captured stably on the substrate surface [24]. As a result, high-performance SERS-based detection cannot be achieved directly. Thus, the design and development of a new multifunctional composite SERS substrate are of great importance. Such a substrate should have high SERS activity while also being capable of selectively capturing and enriching the surface of VOC molecules.

Plasmonic metal surface modification represents an effective strategy for capturing or adsorbing VOCs efficiently [24]. Various modification strategies have been proposed for SERS-based VOC detection, primarily involving chemical-specific adsorption and physical structure capture. Chemical-specific adsorption strategies facilitate the selective capture of specific VOCs, such as organic modification of plasmonic SERS substrates with 4-aminothiophenol (4-ATP) [25], 2,4-dinitrophenylhydrazine (2,4-DNPH) [26], propanethiol [27], and benzenethiol [28], which can specifically capture molecules such as benzaldehyde and benzene [29]. However, modifications performed through this approach are not reusable, and a mixture of target molecules and modifiers can impede the differentiation of spectral patterns. Conversely, the physical capture approach involves the modification of plasmonic SERS substrates with materials having porous structures, such as mesoporous SiO_2_ [30], mesoporous TiO_2_ [31], oxides [32], layered double hydroxide (LDH) [33], and metal-organic framework (MOF) [34,35], which effectively capture gaseous VOC molecules. For instance, Chen et al. prepared Au@ZIF-8 core-shell nanoparticles (NPs) to effectively capture VOCs such as toluene and ethylbenzene by precisely controlling the shell thickness of ZIF-8 [36]. Zhang et al. used a three-dimensional mesoporous silica gel modified with silver nanoparticles for ultra-trace detection of various toxic VOCs, including toluene, benzene, chloroform, and acetone, using enhanced SERS and CERS dual modes [30]. However, coatings such as mesoporous SiO_2_ and TiO_2_ capture VOCs molecules only by their porous structure with low efficiency, resulting in a slow Raman response [25,37]. Moreover, the modified materials have many spectral modes, making it difficult to clearly distinguish target molecules [31,34]. Meanwhile, the lower detection limit reaches only tens or hundreds of ppm, which is inadequate for practical applications.

In this study, we present a straightforward and efficient approach for the rapid and sensitive SERS detection of MACHs VOCs utilizing a bifunctional composite SERS substrate. This bifunctional substrate comprises a copper hydroxide/gold-ordered nanoarray composite, designed and fabricated by the simultaneous preparation of an Au/SiO_2_ reinforcement layer and a porous Cu(OH)_2_ adsorption layer using a gas-liquid interface self-assembly method. The porous Cu(OH)_2_ thin layer on the surface features banding, random, and tight entanglement, forming a network with a large surface area, enabling excellent adsorption performance. The adsorption layer specifically captures gaseous MACHs, including benzaldehyde, benzene, xylene, styrene, and nitrobenzene, among others. The Au/SiO_2_ micro-nano structure at the bottom contains numerous SERS hot spots in the cell gap and on the surface. The Raman characteristic signal of MACHs molecules captured by the adsorption layer can be amplified at a high magnification due to the enhanced layer, leading to the achievement of trace-specific detection. Typically for gaseous benzaldehyde, the combination of the enhanced layer and the adsorption layer not only gives the composite substrate an ultra-low detection limit (less than 500 ppt) and an ultra-fast response time (<20 s), but also the composite substrate is recoverable in SERS response, making it reusable and suitable for widespread application. Overall, this work presents not only a simple method for detecting trace gaseous VOCs using SERS but also a rational material design for the development of VOCs detection substrates.

## 2. Experimental Section

### 2.1. Materials and Reagents

Copper acetate monohydrate (Cu(CH_3_COO)_2_∙H_2_O) and Formamide (CH_3_NO) were obtained from Shanghai Aladdin BioChem Technology Co., Ltd. (Shanghai, China). Benzaldehyde, styrene, nitrobenzene, xylene, toluene, benzene, aldehyde, acetone, ethanol, cyclohexane hydrogen sulfide, aqueous ammonia, N-Butanol, Sodium dodecyl sulfate (SDS) and Rhodamine 6G (R6G) were obtained from Sinopharm Chemical Reagent Co., Ltd. (Shanghai, China). Si (100) wafers were bought from Zhejiang Lijing Silicon Material Co., Ltd. (Quzhou, China). Suspensions of SiO_2_ spheres (150 nm in diameter, 5 wt% dispersion in water) from Huge Biotechnology (Shanghai, China) were used for self-assembling SiO_2_ monolayer templates. Au target (99.999% in purity) used in this study was provided by ZhongNuo Advanced Material (Beijing) Technology Co., Ltd. (Beijing, China). Deionized water, with a resistivity of 18.2 MΩ cm, was obtained from an ultrafilter system (Millipore Milli-Q system, Marlborough, MA, USA). The VOCs gas detection vessel used in the experiment was custom-made. The glassware had been washed with acetone, ethanol, deionized water, and aqua regia before use.

### 2.2. Fabrication of Bifunctional Copper Hydroxide/Gold-Ordered Nanoarray Composites

**Figure 1:**
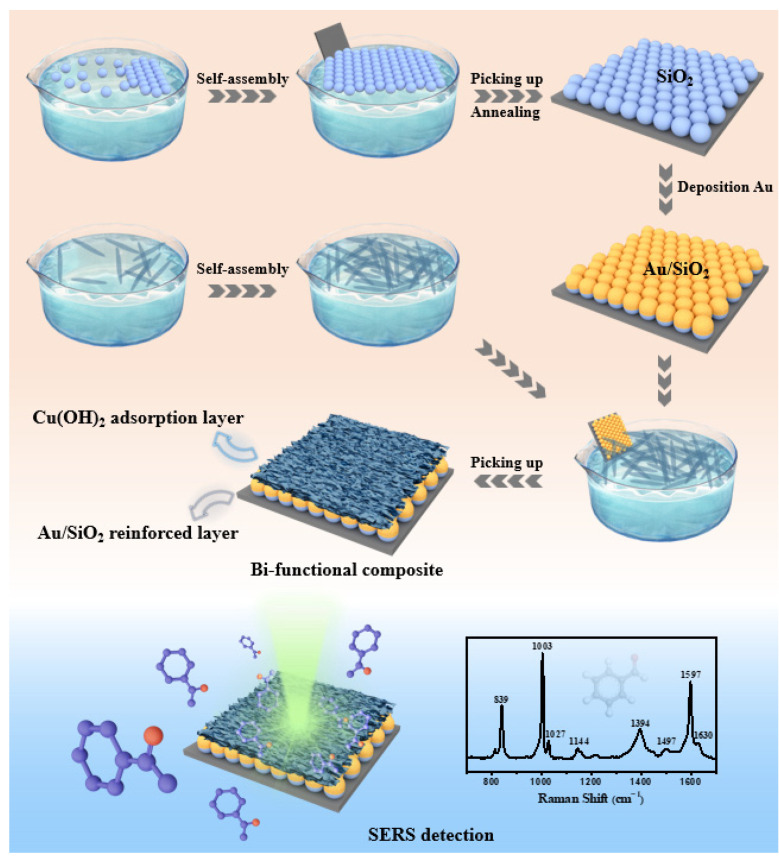
Schematic diagram of the fabrication route for Cu(OH)_2_/Au/SiO_2_ and benzaldehyde detection strategy. The bifunctional copper hydroxide/gold-ordered nanoarray composite was designed and fabricated by a microfluidic assisted gas-liquid interface self-assembly method.

The bifunctional copper hydroxide/gold-ordered nanoarray composite was designed and fabricated via combining a reinforcement layer, which involved depositing Au on the SiO_2_ pellet monolayer template, and a porous copper hydroxide adsorption layer, as schematically illustrated in Figure 1.

An ultra-uniform and tightly packed SiO_2_ pellet monolayer template comprising 150 nm SiO_2_ spheres was prepared on the surface of deionized water via a microfluidic-assisted gas--liquid interface self-assembly method. The template was then transferred onto a clean Si (100) wafer treated with ozone, with 3 × 3 cm in size, following previously reported procedures [38]. Subsequently, the sample was heated at 400 °C for 120 min to obtain a SiO_2_ nanosphere array. To achieve an Au-coated SiO_2_ nanosphere array (Au/SiO_2_), Au-sputtering deposition was carried out at a rate of 11.25 nm/min in equivalent thickness for 8 min using the Q150R plus Sputter Coating System with 30 mA in the deposition current. Using the microfluidic-assisted gas-liquid interface self-assembly method ensures the preparation of a highly uniform and easily controllable Au/SiO_2_ reinforced layer.

A solution containing precursor (80 mM) was obtained by dissolving the copper acetate monohydrate (0.8 g) in a mixture of deionized water (20 mL) and formamide (20 mL). Next, the solution was ultrasonically dispersed, and the vials were sealed with plastic wrap with multiple holes drilled to allow for gas diffusion. The precursor solution vial was placed into a sealed blue-capped bottle (100 mL) containing 2 mL of concentrated ammonium hydroxide solution. After 15 h of diffusion under room temperature, the sediment at the bottom of the vial was washed three times through centrifugation with deionized water (8000 rpm, 3 min) and dried in a vacuum. The simple wet chemical precipitation method is conducive to the synthesis of adsorption materials with a large number of pores, a high specific surface area, and multiple active sites [39].

Finally, a certain amount of precipitates was dissolved in ethanol, ultrasonically cleaned for 30 min, and then mixed evenly. Similarly, a thin film was prepared on the surface of deionized water via a microfluidic-assisted gas--liquid interface self-assembly method, transferred to Au/SiO_2_ reinforced substrate, and then dried at room temperature. A bifunctional copper hydroxide/gold-ordered nanoarray composite was thus obtained. The self-assembly method is advantageous for achieving a uniform and high-quality bifunctional composite.

### 2.3. Characterization

The field-emission scanning electron microscopy (FESEM, Hitachi SU8020, Hitachi Hightech International Trade Co., Ltd., Shanghai, China) and high-resolution transmission electron microscopy (TEM, JEOL JEM-2100, JEOL Science and Trade Co., Ltd., Beijing, China) attached with an energy-dispersive spectrometer (EDS, Oxford Aztec X-Max 80, Oxford Instruments Group, Shanghai, China) were applied to characterize morphology, microstructure and composition analyses. The X-ray diffraction (XRD) measurements of the products were carried out using Cu Kα1 radiation (λ = 1.5406 Å) on an X-ray diffractometer (the Philips X’Pert). The X-ray photoelectron spectroscopy (XPS) measurements were performed on a Thermo ESCALAB 250XI (Thermo Fisher Scientific, Waltham, MA, America) photoelectron spectrometer that operated at an acceleration voltage of 15 kV and a current of 10 mA, with binding energies calibrated using the C 1 s orbital (284.8 eV). Thermogravimetric analysis (TGA/DSC) was carried out using a Mettler-Toledo TGA/DSC 3+. The measurements were performed by heating the sample from 25 °C to 300 °C at 1 °C∙min^−1^ under air flow. The N_2_ sorption isotherms were measured on a specific surface area and porosity analyzer (Micromeritics, ASAP 2460 3.01, Micromeritics Instrument Co., Ltd., Shanghai, China). The degassing temperature and degassing time of samples were 120 °C and 6 h, respectively. The simulation of the EM enhancement effect around Au/SiO_2_ under 785 nm wavelength laser excitation was performed in COMSOL 6.0 software.

### 2.4. Raman Spectral Measurements

Raman spectroscopy measurements were conducted on a custom detection platform equipped with a quartz window (3 cm × 3 cm), which was used as a channel for incident light and Raman light, as schematically illustrated in Scheme S1. A SERS composite was inserted into the detection platform, and a specific quantity of gaseous MACHs (such as benzaldehyde, styrene, nitrobenzene, benzene, xylene, toluene, etc.) was injected. The gas concentration was calculated using the injection amount and the platform volume. In situ Raman spectroscopy measurements were taken of the SERS composite surface using a portable Raman spectrometer (BWS415-785S, B&W TEK Opto-electronics, Co., Ltd., Shanghai, China) with excitation at 785 nm. The laser spot on SERS composites was 5 µm. Laser power was set at 20 mW for all Raman spectroscopy measurements with a 10 s integration time. In-situ Raman spectra measurements were obtained at an average room temperature, and a relative humidity of 35% was maintained within the device.

## 3. Results and Discussion

### 3.1. Bifunctional Copper Hydroxide/Gold Ordered Nanoarray Composites

Bifunctional copper hydroxide/gold-ordered nanoarray composites were directly fabricated via the microfluidic-assisted gas-liquid interface self-assembly method and systematic experiments were subsequently conducted for structure, morphology, composition, and SERS property evaluations.

#### 3.1.1. The Au/SiO_2_ Reinforcement Layer

A tightly packed 150 nm SiO_2_ monolayer with a large area (Figure S1a) was prepared on a Si (100) wafer via a microfluidic-assisted gas-liquid interface self-assembly method. The SiO_2_ spheres were hexagonally arranged in close. This method provided precise flow- rate control and minimized anthropogenic variability, thereby solving the issue of multi-layered spheres. The resulting SiO_2_ pellet monolayer offered the advantage of a large area single layer, as shown in Figure S1b.

**Figure 2:**
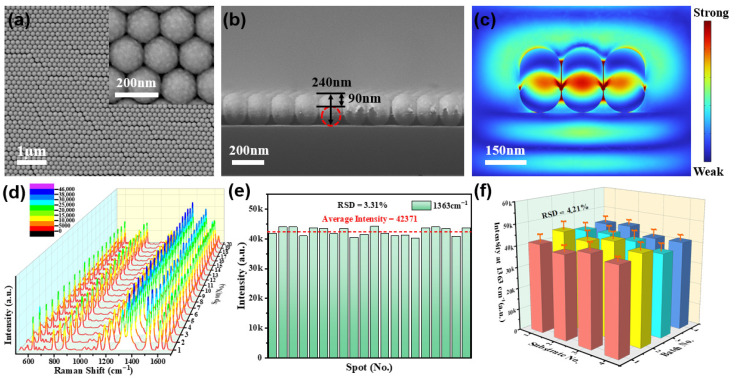
(**a**) Top-view and (**b**) cross-section FESEM images of the 90 nm Au-covered SiO_2_ nanoarray. The inset in (**a**) shows a magnified view of a local area. (**c**) The FDTD theoretical simulation of Au-covered SiO_2_ nanoarray under 785 nm wavelength laser excitation. (**d**) Raman spectra collected from 20 randomly selected points on the 10^−5^ M R6G-soaked Au/SiO_2_ substrate. (**e**) Histogram of the peak intensities at 1363 cm^−1^ [data from (**d**)]. (**f**) Histogram of the R6G peak intensities at 1363 cm^−1^ for 16 Au/SiO_2_ samples (4 batches) after immersing them in 10 mL of 10^−5^ M R6G solution for 1 h.

After Au was deposited with 90 nm in equivalent thickness (Figure S1c) on the SiO_2_ sphere nanoarray, the Au-wrapped SiO_2_ sphere nanoarray (Au/SiO_2_) was prepared, as shown in Figure 2a. The resulting nanoarray consisted of nearly spherical Au films that were approximately 150 nm in size, which had rough surfaces and multiple protrusions (8–15 nm), as illustrated in the inset of Figure 2a. Cross-sectional observations revealed that the SiO_2_ spheres were coated with a 90 nm thick Au film (Figure 2b). The finite difference time domain (FDTD) theoretical simulation demonstrated that the gap and rough particle surface of the nanoarray exhibited an extremely strong electric field enhancement, contributing significantly to SERS intensity. Specifically, an electric field enhancement region of approximately 100 nm existed above the nanoarray, as shown in Figure 2c.

The Au/SiO_2_ substrate demonstrated a high degree of structural homogeneity at the micron scale, which can be attributed to the high quality of the SiO_2_ monolayer and the small size (150 nm) of the spheres. This characteristic resulted in highly repeatable Raman measurements, as illustrated in Figure 2d. Twenty random sites on the 1 cm × 1 cm substrate were tested by immersing the spheres in 10 mL of 10^−5^ M R6G solution for 1 h, producing a small Relative Standard Deviation (RSD) of characteristic peak intensities (generally, <3.31% for the peak at 1363 cm^−1^) (indicated in Figure 2e), demonstrating excellent signal reproducibility. Reproducibility between batches was also evaluated. Using four batches of the Au/SiO_2_ substrates (16 substrates totally), all prepared under the same conditions, the RSD of the R6G peak intensities at 1363 cm^−1^ across all 16 substrates was 4.21%, as illustrated in Figure 2f. Overall, the results demonstrated that a microfluidic-assisted template and deposition can achieve superior reproducibility between batches. In addition, an optimal Au deposition thickness was found to have an impact on SERS activity. The maximum SERS activity was observed at an Au deposition thickness of approximately 90 nm, while overly thick or thin deposition led to a significant reduction in SERS activity, as illustrated in Figure S1d.

#### 3.1.2. The Cu(OH)_2_ Adsorption Layer

**Figure 3:**
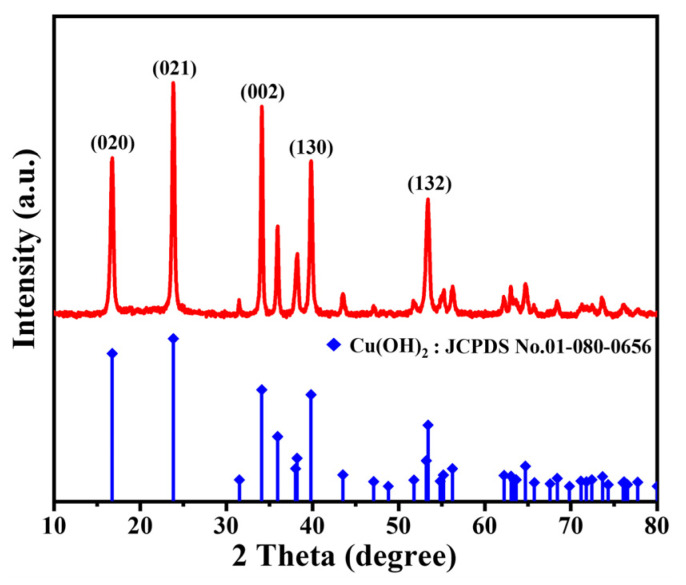
Phase structure of the as-prepared adsorption layer material. XRD pattern. The line spectra are the standard patterns of Cu(OH)_2_ (PDF No. 80−0656, respectively, JCPDS).

The X-ray diffraction (XRD) pattern of the adsorption layer material, prepared via the wet chemical precipitation method, is presented in Figure 3. The pattern displays distinct peaks which can be readily indexed to Cu(OH)_2_, using the Powder Diffraction File (PDF) No. 80-0656 from the Joint Committee on Powder Diffraction Standards (JCPDS). The most prominent peaks of the XRD pattern at 16.7°, 23.8°, and 34.1° correspond to the (020), (021), and (002) lattice planes of orthotropic Cu(OH)_2_, respectively.

**Figure 4:**
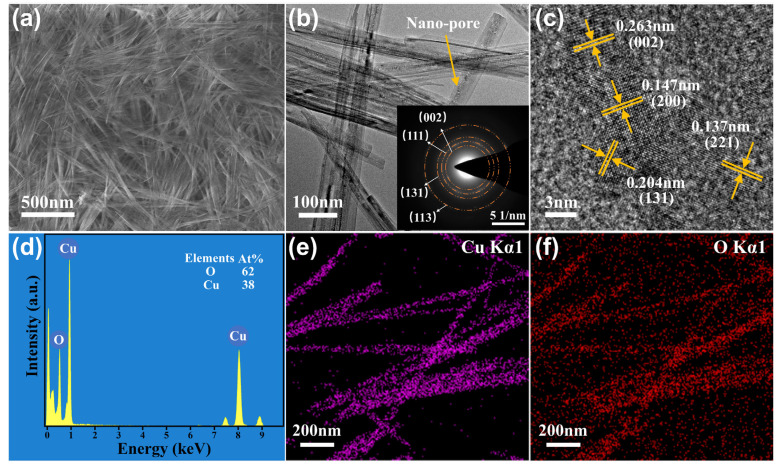
Microstructure and surface state analyses for the Cu(OH)_2_ adsorption layer. (**a**) Top-view FESEM image. (**b**) Typical TEM observation, with the corresponding SAED pattern inset. (**c**) HRTEM image. (**d**) EDS and (**e**,**f**) elemental mapping demonstrations.

The Cu(OH)_2_ thin film obtained through the gas-liquid interface self-assembly method comprises closely-knitted and randomly-tangled Cu(OH)_2_ nanoribbons, consequently forming a network structure with a large surface area suitable for highly sensitive gas adsorption detection, as typically illustrated in Figure 4a. Furthermore, upon microstructural examination of the Cu(OH)_2_ adsorption layer, TEM observation revealed numerous minuscule pores (of only a few nanometers) in the band, measuring 30–80 nm in width and less than 5 nm in thickness, as illustrated in Figure 4b. Notably, the corresponding SAED pattern (inset in Figure 4b) affirms that the band has various lattice surfaces of the orthotropic Cu(OH)_2_, notably (002), (111), (131), and (113), hence depicting its polycrystalline structure, albeit with a certain single-crystal tendency, as supported by observations of weak diffraction rings. Furthermore, following high-resolution (HR)-TEM analysis, it became evident that the lattice fringes on the Cu(OH)_2_ nanoribbons corresponded to the Cu(OH)_2_ crystal’s (002), (200), (131), and (221) planes, with a lattice fringes spacing of 0.263, 0.147, 0.204, and 0.137 nm, respectively, as presented in Figure 4c, which matched the data of Cu(OH)_2_ in JCPDS card No. 80-0656. The EDS analysis and corresponding element mappings indicate that the porous adsorption layer is composed of Cu and O with the atomic ratio of 0.6:1 (Figure 4d–f).

#### 3.1.3. Bifunctional Composites

**Figure 5:**
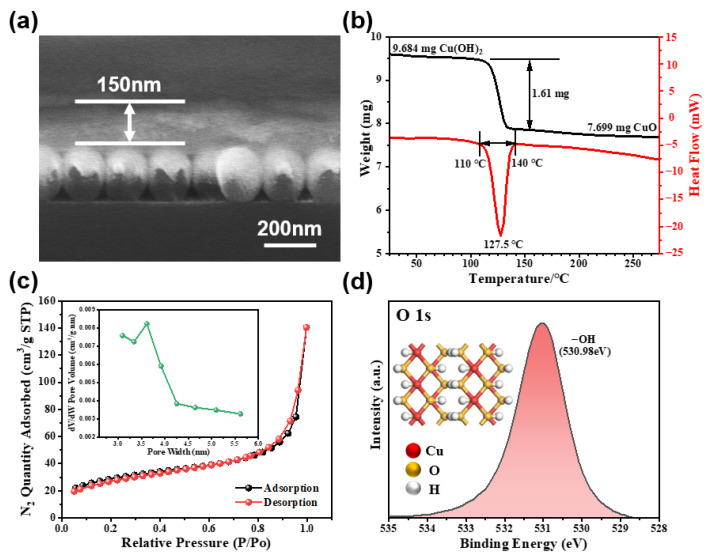
(**a**) FESEM image of the Cu(OH)_2_/Au/SiO_2_ (cross-sectional view). (**b**) The TGA/DSC curve of the Cu(OH)_2_ adsorption layer. (**c**) The N_2_ sorption isotherms of the Cu(OH)_2_ adsorption layer, with the pore size distribution presented in the inset. (**d**) Binding energy spectra of O 1 s.

The Cu(OH)_2_/Au/SiO_2_ composites were fabricated using the method depicted in Figure 1. Cu(OH)_2_ self-assembled thin films were deposited onto an Au/SiO_2_ substrate, resulting in an adsorption layer with a thickness of 150 nm, as shown in Figure 5a. The XRD pattern and EDS analysis also proved the successful preparation of the composites, as illustrated in Figure S2. Especially, it is worth mentioning that the thickness of the Cu(OH)_2_ adsorption layer can be conveniently adjusted by simply altering the number of times that Cu(OH)_2_ self-assembled films are picked up by the Au/SiO_2_ substrate. As shown in Figure S3, the thickness of the Cu(OH)_2_ adsorption layer can be increased to 300 nm when the number of film depositions reaches six.

Copper hydroxide, an unstable copper compound, is recognized for its propensity to decompose at elevated temperatures [40]. To assess the Cu(OH)_2_ adsorption layer’s thermal stability in the composites, we conducted a thermal stability study. Figure 5b shows the TGA/DSC curve of the Cu(OH)_2_ adsorption layer. The TG analysis indicates that Cu(OH)_2_ undergoes decomposition into CuO and H_2_O at 110 °C, which elicits a potent endothermic peak in the heat flow curve. Notably, the gas detection temperature is typically below 110 °C. Therefore, Cu(OH)_2_ is relatively stable within this temperature range, making it suitable for gas detection applications.

The isothermal N_2_ sorption measurements were carried out on the porous Cu(OH)_2_ adsorption layer coated on the composites as illustrated in Figure 5c. The results indicate that Cu(OH)_2_ exhibits an remarkably large specific surface area, estimated to be 104.7799 m^2^∙g^−1^, which is extremely favorable for the adsorption of gases. Analysis of the pore size shows that most pores in the Cu(OH)_2_ coating have a diameter of approximately 3.6 nm. Gas adsorption relies heavily on pore distribution since pores that are excessively small may trap gases, leading to a swift response and sluggish sensor recovery. Conversely, excessively large pores will easily lead to defects, filling with impurity, and agglomeration, resulting in poor sensor selectivity [41].

To further analyze the surface chemistry of the Cu(OH)_2_/Au/SiO_2_ composites, XPS measurements were conducted. As shown in Figure S4a, the full XPS spectrum of the composites displays the presence of elements Au, Cu, O, and C, with element C serving as a reference. Figure S4b presents the binding energy spectrum of Cu 2p, with the peaks at 934.38 and 954.28 eV assigned to Cu 2p_3/2_ and Cu 2p_1/2_ in Cu(OH)_2_, respectively, and an energy gap of 19.9 eV between the two peaks. In addition, there are Cu 2p_3/2_ and Cu 2p_1/2_ jitter satellite peaks at 943.4 and 963.1 eV. It is explicitly shown that the valence state of Cu^2+^ is consistent with the standard Cu value of Cu(OH)_2_ reported in the literature [42]. Figure 5d shows the binding energy spectrum of O 1s, which demonstrates a broad peak at 530.98 eV from oxygen in (−OH) groups, indicating a high concentration of hydroxyl groups in the Cu(OH)_2_. This abundance of hydroxyl groups facilitates the electrostatic adsorption of MACHs gas molecules [24].

### 3.2. SERS-Based Detection of Trace MACHs

The above porous Cu(OH)_2_ adsorption layer covered (150 nm in thickness) composite, the bare Au/SiO_2_, and Cu(OH)_2_-covered Au/Si were used as the SERS substrates to study their responses to the trace MACHs.

#### 3.2.1. The Spectral Patterns

**Figure 6:**
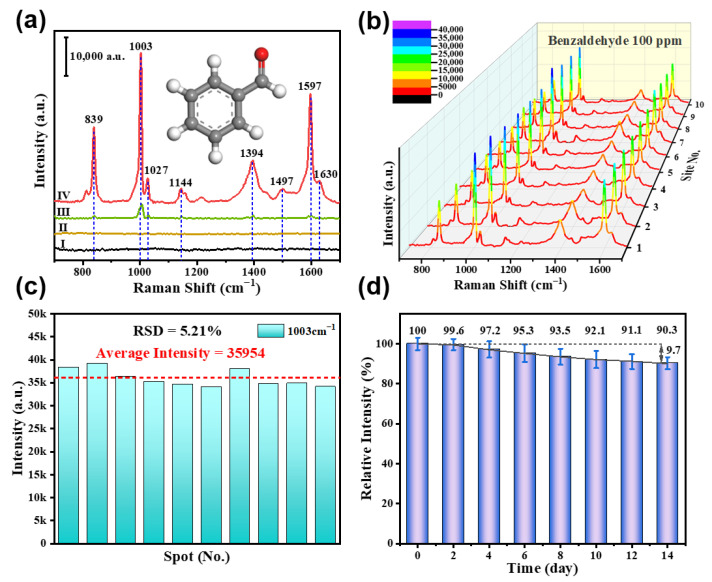
(**a**) Raman spectra before (I) and after (II, III, and IV) exposure to 100 ppm benzaldehyde-containing air for 2 min. I and IV are for the composites, II is for the bare Au/SiO_2_ substrate, and III is for the Cu(OH)_2_-covered Au/Si substrate. (**b**) Raman spectra collected from 10 random spots after exposure to 100 ppm benzaldehyde-containing air for 2 min. (**c**) Histogram of the peak intensities at 1003 cm^−1^ [The data are from (**b**)], with RSD indicating the relative standard deviation. (**d**) Histogram of the Raman peak intensities at 1003 cm^−1^ for the 150 nm Cu(OH)_2_-covered Au/SiO_2_ substrate after various durations of storage in air and exposure to 100 ppm benzaldehyde-containing air for 2 min.

To assess the efficiency of the Cu(OH)_2_/Au/SiO_2_ composites in capturing MACHs, we used benzaldehyde as the target molecules. Prior to exposure to the benzaldehyde-containing air, no response was observed for the bare Au/SiO_2_ substrate and the Cu(OH)_2_-covered Au/Si substrate, as illustrated in curve Ⅰ of Figure 6a. However, upon exposure to 100 ppm benzaldehyde vapors, the composites substrate exhibited a strong Raman response, as illustrated in curve Ⅳ of Figure 6a. The resulting Raman spectrum, as shown in Table S1, showed distinct Raman peaks at 839, 1003, 1027, 1144, 1394, 1497, 1597, and 1630 cm^−1^, assigned to Φ1 + δ(CCO) + ν(C-C), Φ12, Φ18a, Φ13, ν_s_(OCO), Φ19a, Φ8a, and ν(C=O), respectively [43]. However, the bare Au/SiO_2_ substrate shows no response after being exposed to 100 ppm benzaldehyde-containing air, indicating the difficulty of plasma SERS substrates in capturing and detecting gaseous MACHs (curve Ⅱ of Figure 6a). Additionally, the Cu(OH)_2_-covered Au/Si substrate, with no array SERS enhancement, demonstrated a weak Raman signal upon benzaldehyde exposure, much weaker and slightly shifted compared to the exposed composites (curve III in Figure 6a). The resulting weak signal may be attributed to the strongly physical enrichment of benzaldehyde molecules or the chemisorption of molecules due to the porous structure of Cu(OH)_2_. These results highlight the important role of nanoarray structures and the synergistic effect of Cu(OH)_2_ and Au components in enhancing local electromagnetic fields and the gas adsorption of substrates.

#### 3.2.2. The Measurement Reproducibility and Stability of the Composites

The bifunctional Cu(OH)_2_/Au/SiO_2_ composites exhibit excellent signal reproducibility and time stability. Raman spectra from 10 randomly selected locations on a composite substrate were recorded after being exposed to 100 ppm benzaldehyde-containing air for 2 min, as illustrated in Figure 6b. In general, the peak intensities measured at 1003 cm^−1^ have a relative standard deviation (RSD) of less than 6% (Figure 6c). In addition, Figure 6d depicts the Raman peak intensities measured at 1003 cm^−1^ for the composite substrate after being stored in air for various periods and then exposed to 100 ppm benzaldehyde-containing air for 2 min. The results display reasonably good stability for a minimum of 2 weeks. In fact, the composite substrates can maintain long-term stability if they are vacuum-packed before use. The evidence presented demonstrates that the Cu(OH)_2_-covered Au/SiO_2_ is an excellent SERS substrate for detecting MACHs VOCs.

#### 3.2.3. Influence of Cu(OH)_2_ Covering Adsorption Layer Thickness

**Figure 7:**
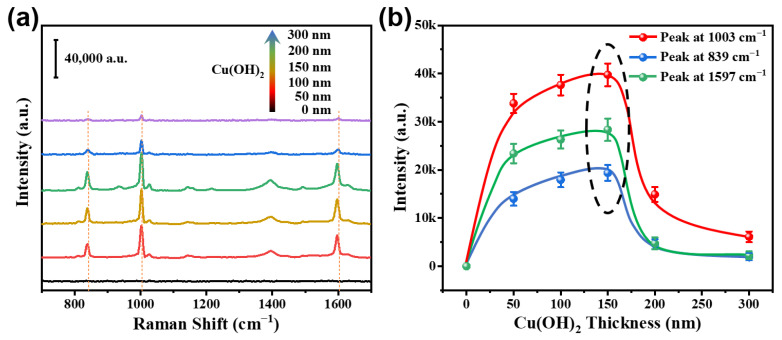
Raman spectral for the different Cu(OH)_2_ covering adsorption layer thickness. (**a**) Raman spectra of the composites with different Cu(OH)_2_ covered adsorption layer thicknesses, after exposure to 100 ppm benzaldehyde-containing air for 2 min. (**b**) Main Raman peak intensities of benzaldehyde as a function of Cu(OH)_2_ covered adsorption layer thickness [data from (**a**)].

The thickness of the Cu(OH)_2_ covering the adsorption layer is a crucial factor that impacts the SERS response of the composite substrate to gaseous MACHs VOCs. Figure 7a shows the Raman spectra of the 100 ppm benzaldehyde-containing air-exposed Cu(OH)_2_-covered Au/SiO_2_ composite substrate vary with the thicknesses of different Cu(OH)_2_ adsorption layers. According to our experimental findings, the response of the composite substrate is favorable at a thickness of 50 nm. Additionally, within the thickness range of 50–150 nm, we have observed a gradual increase in response, followed by a sudden decrease. The substrate with a Cu(OH)_2_ covering thickness of about 150 nm shows the highest response to benzaldehyde, while thicker or thinner Cu(OH)_2_ covering layers lead to smaller responses, as clearly demonstrated in Figure 7b. Therefore, the Cu(OH)_2_-covered Au/SiO_2_ composite substrate with a Cu(OH)_2_ layer thickness of 150 nm was used for further work.

#### 3.2.4. Dependence on Gas Concentration

**Figure 8:**
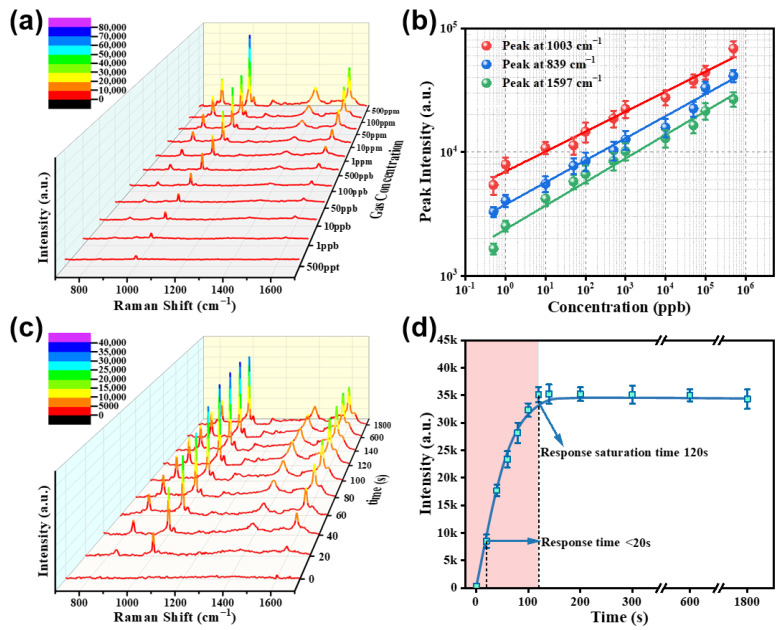
Raman spectral dependency of the Cu(OH)_2_/Au/SiO_2_ composites exposed to benzaldehyde gas as a function of concentration and exposure time. (**a**) Raman spectra of the composites after exposure to benzaldehyde-containing air with varying concentrations for 2 min. (**b**) Plots of main Raman peak intensity versus benzaldehyde gas concentration on a logarithmic scale [data from (**a**)]. (**c**) Raman spectra of the composites after exposure to 100 ppm benzaldehyde gas for varying time intervals. (**d**) Intensity of the main Raman peak at 1003 cm^−1^ as a function of exposure time [data from (**c**)].

Furthermore, the dependence of the Raman spectra on the concentration of gaseous benzaldehyde was investigated for the 150 nm Cu(OH)_2_-covered Au/SiO_2_ composite substrate. Obviously, the gas concentration has a significant effect on the Raman response. Figure 8a displays the Raman spectra of the composite substrate exposed to benzaldehyde-containing air at different concentrations for 2 min. The intensity of the peaks increased as the concentration of gaseous benzaldehyde increased, and the substrate demonstrated exceptional sensitivity, detecting concentrations as low as 500 ppt or less. It is crucial to note that there are linear correlations between the concentration (C) and the intensity of the primary peak (I) (in logarithmic scale) over a vast range of 500 ppt to 500 ppm, or

(1) $$LogI=A_{1}+B_{1}\cdot LogC$$

where the parameters *A*_1_ and *B*_1_ are the constants. Figure 8b gives the double logarithmic plot of *I* versus *C* for the main peak at 1003, 839, and 1597 cm^−1^, which exhibit excellent linear relationships. The values of *A*_1_ and *B*_1_ were determined by linear fitting to be 3.84, 3.37, 3.57 and 0.16, 0.19, 0.17, respectively. Such linear relationships enable quantitative analysis of SERS measurement results.

#### 3.2.5. Dependence on Exposure Time

The time-dependent behavior of Raman spectra upon exposure to gaseous benzaldehyde was investigated, and the results are presented in Figure 8c. The in situ Raman spectra of the composite substrate were collected after exposure to 100 ppm benzaldehyde-containing air for various time intervals. The composite substrate exhibited remarkable sensitivity to gaseous benzaldehyde, showing a response within 20 s. The main peak intensity at 1003 cm^−1^ increased almost linearly with the increase of exposure time, reaching a saturation value after exposure for 2 min. Further extension of the exposure time did not result in significant changes in Raman signals, indicating the attainment of equilibrium adsorption of benzaldehyde on the composite substrate, as depicted in Figure 8d. These findings suggest that the composite substrate can be utilized for real-time monitoring of VOCs.

#### 3.2.6. Spectral Recoverability and Recyclability

**Figure 9:**
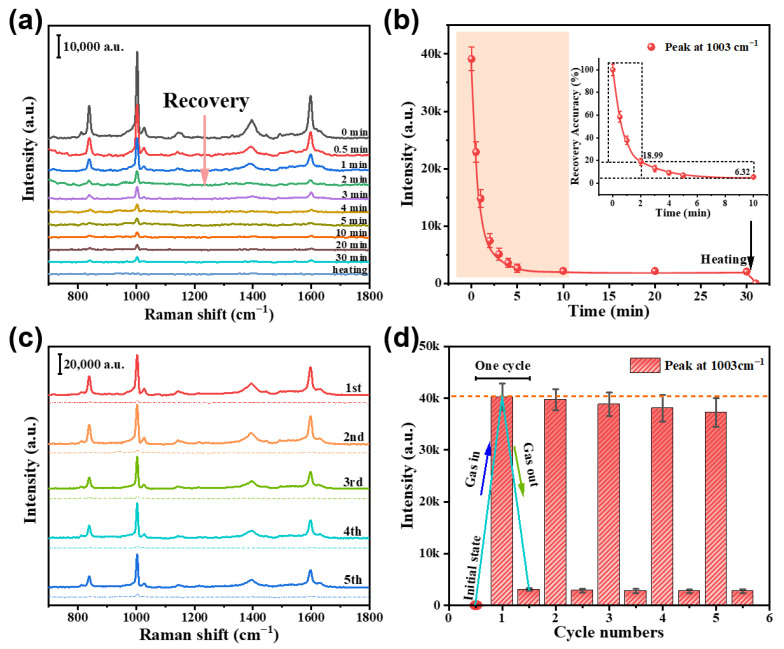
The Raman spectral recoverability and recyclability for the Cu(OH)_2_/Au/SiO_2_ composites. (**a**) The Raman spectra after venting (or opening the lid of the test device) for different time-intervals for the composites exposed to 100 ppm benzaldehyde-containing air for 2 min. The bottom curve is the spectrum after venting for 30 min and then heating at 60 °C for 20 min. (**b**) Peak intensity at 1003 cm^−1^ for the 2-min exposed composites as outgassing time elapses. The inset shows a magnified figure in the time range of 0–10 min based on the data from (**a**). (**c**) The Raman spectra after alternate gas-in for 2 min (solid lines) and gas-out for 10 min (dashed lines) (gas-in: 100 ppm benzaldehyde. 5 cycles in total). (**d**) The Raman peak intensities at 1003 cm^−1^ for the composites after alternate gas-in and gas-out cycles [the data from (**c**)].

The recyclability of the composite substrate was evaluated by desorbing the gaseous benzaldehyde from the homemade device. Figure 9a presents the in situ Raman spectra after a 2 min exposure to 100 ppm benzaldehyde-containing air, recorded as a function of time following the venting process via opening the device lid. The peak intensity of the composite substrate at 1003 cm^−1^ after venting durations is shown in Figure 9b. The signal intensity rapidly dropped to 18.99% of its saturation value within 2 min, followed by a gradual decline until 10 min, at which an extended duration led to a further decrease in the intensity value to 6.32%. After 30 min of venting, the signal intensity remained at approximately 6.32% of the saturation value, i.e., the Raman response after venting could mostly (~90–95%) recover, but not fully. This indicates that although most physically adsorbed molecules were desorbed, a small fraction of chemisorbed benzaldehyde molecules on the composite substrate were irreversibly bound, resulting in incomplete recovery of the Raman signal. However, the Raman signal could be eliminated by subsequent heating at 60 °C for 20 min.

The performance of the composite substrate was also evaluated under cyclic exposure to benzaldehyde by injecting 100 ppm benzaldehyde for a duration of 2 min and then alternating with a 10-min venting period. The in situ Raman spectra of the composite substrate in the test device are shown in Figure 9c, and the peak intensities at 1003 cm^−1^ for each cycle test are presented in Figure 9d. The results show an almost recoverable Raman signal with only a slight decrease, while the total intensity consistently remained at approximately 5–10%.

#### 3.2.7. Multiplex VOCs and Selectivity

**Figure 10:**
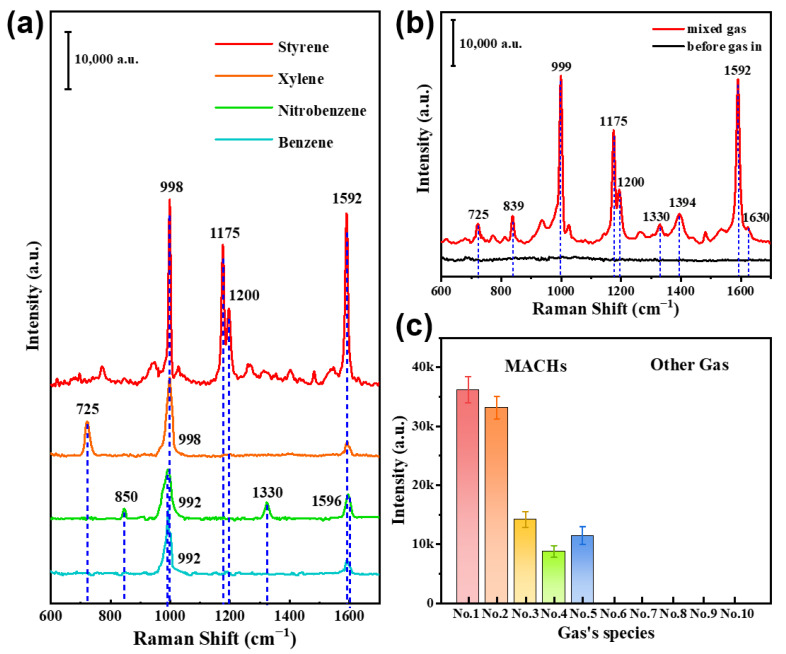
Molecular distinguishability of the Cu(OH)_2_/Au/SiO_2_ composites. (**a**) Raman spectra of the composites after exposure to 100 ppm gaseous MACHs (styrene, xylene, nitrobenzene, and benzene) for 2 min. (**b**) Raman spectra of the composites before and after exposure to the mixed gaseous MACHs for 2 min, constituting 100 ppm styrene, 100 ppm xylene, 100 ppm nitrobenzene and 100 ppm benzaldehyde. (**c**) Histograms of the main peak intensities for the composites after exposure to different VOCs (100 ppm) for 2 min [represented by peaks around 1000 cm^−1^, the data from (**a**) for MACHs]. Nos. 1–9 correspond to the MACHs (benzaldehyde, styrene, xylene, nitrobenzene, and benzene) and the non-benzene hazardous gases (hydrogen sulfide, ethanol, acetone, formaldehyde, and cyclohexane), respectively.

The composite substrate exhibited a significant response not only to benzaldehyde but also to other gaseous MACHs, namely styrene, xylene, nitrobenzene, and benzene, as illustrated in Figure 10a. The MACHs exhibit distinct Raman spectra, which are listed in Table S2, including their characteristic peak positions and assignments. By analyzing these characteristic peak positions, the MACHs can be easily identified using the composite substrates. Significantly, the composite substrate can detect and distinguish these MACHs even when they are present as a mixture. Figure 10b displays the Raman spectra corresponding to a concentration ratio of benzaldehyde, styrene, xylene, and nitrobenzene of 1:1:1:1. The peak at 999 cm^−1^ is the superposition of the benzene ring stretching vibrations of these four MACHs molecules. According to Table S2, the composite substrates can detect the characteristic peaks of benzaldehyde (839 and 1630 cm^−1^), styrene (1175 and 1200 cm^−1^), xylene (725 cm^−1^) and nitrobenzene (1330 cm^−1^), as well as the co-contribution peak at 999 cm^−1^.

Additionally, the composite substrate’s selectivity to MACHs was also evaluated. Non-benzene gases, including acetone, hydrogen sulfide, ethanol, formaldehyde, and cyclohexane, did not yield any SERS response on the composites, even at high concentrations (1000 ppm). Figure 10c shows peak intensities for the composites exposed to the MACHs and other non-benzene hazardous gases at a concentration of 100 ppm, showing the high selectivity of the composites to MACHs.

In summary, our results demonstrate that SERS using the composites presented in this study offers several advantages over previously reported methods for detecting gaseous MACHs, including reduced false alarm rates (or improved identifiability), faster response times, increased sensitivity, and greater measurement portability. These findings are summarized in Table S3.

## 4. Conclusions

In summary, we designed and fabricated a rapid and sensitive SERS method for MACHs detection using a bifunctional substrate, which consists of an Au/SiO_2_ enhancement layer and a porous Cu(OH)_2_ adsorption layer of appropriate thickness (150 nm), which were prepared via microfluidic-assisted gas-liquid interface self-assembly. Due to the excellent gas adsorption performance of the Cu(OH)_2_ and the pronounced surface plasmon resonance (SPR) effect of the Au/SiO_2_, the composite substrate can be well used to monitor the changes in benzaldehyde through time-varying SERS spectroscopy, and to achieve track-specific identification of various volatile organic gases, such as benzene, xylene, styrene, and nitrobenzene. This composite substrate exhibits a rapid and sensitive Raman response to MACHs VOCs. Specifically, for gaseous benzaldehyde, the combination of the enhancement layer and the adsorption layer not only endows the substrate with an ultra-low detection limit (less than 500 ppt) and an ultra-fast response time (<20 s), but also the composite substrate is recoverable and repeatable in the SERS response use. This study not only provides a simple and effective method for the rapid and sensitive detection of trace gaseous VOCs using SERS, but also has great significance for the rational design of SERS substrates for the detection of other VOCs.

## Data Availability

No new data were created or analyzed in this study. Data sharing is not applicable to this article.

## Supplementary Materials

The following supporting information can be downloaded at: https://www.mdpi.com/article/10.3390/nano13132016/s1. Table S1: Vibrational wavenumbers (cm^−1^) and assignments of the SERS spectra of Benzaldehyde. Table S2: The characteristic peaks and their assignments for the SERS spectra of other typical MACHs. Table S3: Comparison of various methods for detecting trace gaseous MACHs. Scheme S1: Schematic illustration of the setup for the in-situ Raman spectral measurements of MACHs. Figure S1: The FESEM image of (a) the SiO_2_ colloidal monolayer on a Si wafer. (b) Cross-section view of the SiO_2_ colloidal monolayer. (c) The linear plot of the Au deposition thickness versus deposition time. (d) The Raman spectra of the R6G-soaked Au/SiO_2_ with different Au deposition thicknesses. The inset of (d) is the peak intensity at 1363 cm^−1^ versus Au deposition thickness. Figure S2: XRD pattern (a) and EDS elemental mapping demonstrations (b) of the Copper Hydroxide/Gold- Ordered Nanoarray Composites. Figure S3: The covering-thickness control of the Cu(OH)_2_-covered Au/SiO_2_. (a–d) The FESEM images of the Cu(OH)_2_-covered Au/SiO_2_ with different thicknesses of Cu(OH)_2_ covering layers. The insets are the corresponding cross-sectional views. (e) The Cu(OH)_2_ covering thickness versus the bailing film times. Figure S4: (a) The XPS measurement of the Cu(OH)_2_-covered Au/SiO_2_. (b) Binding energy spectra of Cu 2p. References [30,36,44,45,46,47,48,49,50,51,52] are cited in the supplementary materials.
