# Supplementary material for: Self-Assembled Bifunctional Copper Hydroxide/Gold-Ordered Nanoarray Composites for Fast, Sensitive, and Recyclable SERS Detection of Hazardous Benzene Vapors
Source: Nanomaterials (Basel). 2023 Jul 6;13(13):2016. doi: 10.3390/nano13132016 (PMC10343417; doi:10.3390/nano13132016)
Supplement: Supplementary file 1 [file nanomaterials-13-02016-s001.zip › nanomaterials-2477550-supplementary.pdf]

Self-Assembled Bifunctional Copper Hydroxide/Gold-Ordered Nanoarray Composites for Fast, Sensitive, and Recyclable SERS Detection of Hazardous Benzene Vapors

Yanyan Lu [1,2], Xuzhou Yuan [3,*], Cuiping Jia [4], Biao Lei [1,2], Hongwen Zhang [1,2,*], Zhipeng Zhao [1,2], Shuyi Zhu [1,2],
Qian Zhao [1] and Weiping Cai [1,2]

[1] Key Laboratory of Materials Physics, Anhui Key Laboratory of Nanomaterials and Nanotechnology,
Institute of Solid State Physics, HFIPS, Chinese Academy of Sciences, Hefei 230031, China; sa21168128@mail.ustc.edu.cn (Y.L.); lbiao@issp.ac.cn (B.L.); zpzhao@issp.ac.cn (Z.Z.);
zhushuyi@issp.ac.cn (S.Z.); zhaoqian@issp.ac.cn (Q.Z.); wpcai@issp.ac.cn (W.C.)
[2] Science Island Branch of Graduate School, University of Science and Technology of China,
Hefei 230026, China
[3] Shandong Hengcheng Testing Technology Co., Ltd., Yantai 261400, China
[4] School of of Economics and Management (SEM), Weifang University of Science and Technology, Weifang 262700, China; jiacuiping2023@126.com
* Correspondence: hctyuan@163.com (X.Y.); hwzhang@issp.ac.cn (H.Z.)

Table S1. Vibrational wavenumbers (cm^{-1}) and assignments of the SERS spectra of Benzaldehyde.

Benzaldehyde (SERS)	
**Raman shift (cm^{-1})**	**Assignment [a]**
839	Φ1 + δ(CCO) [b] + ν(C-C)
1003	Φ12
1027	Φ18a
1144	Φ13
1394	ν_s(OCO)
1423	Φ14/3
1497	Φ19a
1597	Φ8a
1630	ν(C=O) [b]

Notes:
a: The Wilson notation is used for marking the aromatic ring normal modes (Φ), ν - stretching, δ - in-plane deformation. the subscript s refer to the symmetric mode.
b: δ(CCO) and ν(C=O) for the aldehyde.

Table S2. The characteristic peaks and their assignments for the SERS spectra of other typical benzene-related VOCs.

Species of B-VOCs	Raman shift (cm^{-1})	Assignment
**Styrene**	998	benzene ring breathing vibration[S1]
	1175	Stretching vibration between benzene ring and carbon atom [S1]
	1200	Asymmetric stretching vibration between benzene ring and carbon atom [S1]
	1592	C=C stretching and bending vibration of benzene ring [S1]
**Xylene**	725	benzene ring, $-CH_3$ stretching vibration [S2]
	998	benzene ring breathing vibration [S2]
	1592	C=C stretching and bending vibration of aromatic ring [S2]
**Nitrobenzene**	850	benzene ring bending vibration [S3]
	992	benzene ring breathing vibration [S3]
	1330	nitro group stretching vibration [S3]
	1596	C=C stretching and bending vibration of aromatic ring [S3]
**Benzene**	992	benzene ring breathing vibration [S4]

Table S3. Comparison of various methods for detecting trace gaseous benzene-related VOCs.

Techniques (Materials)	Identifiability	Detection limit	Response time	Recyclability	Portability	Reference
**GC-MS**	Good	100 ppt	/	/	No	[S5]
**CRS (ZnO/WO_3[a])**	Poor	100 ppb	0.7 s	Yes	Yes	[S6]
**Chemiluminescence (Y_2O_3)**	Poor	400 ppm	40 s	No	Yes	[S7]
**CM p-n (SnO_2-NiO_x/Cu[b])**	Good	10 ppb	/	No	Yes	[S8]
**SERS (Ag@SiO_2[c])**	Good	68 ppm	/	No	Yes	[S4]
**SERS (Au@ZIF[d])**	Good	/	20 min	Yes	Yes	[S9]
**CRS+SERS (Au@SnO_2[e])**	Good	10 ppm	5 s	/	No	[S10]
**CRS+SERS (Au@SnO_2[f])**	Good	4 ppm	200 s	/	No	[S11]
**SERS ($Cu(OH)_2$@Au/SiO_2[g])**	Good	<500 ppt	<20 s	Yes	Yes	This work

Notes:
GC-MS: Gas chromatography-mass spectrometry;
CRS: Chemical resistance sensor;
CM p-n: Chemical mechanism p-n semiconductor heterostructure.

a: Two-dimensional honeycomb-like ordered porous ZnO/WO_3 sensing matrixes.
b: Novel sponge-like Cu-doping SnO_2-NiO p-n semiconductor heterostructure (SnO_2-NiO_x/Cu).
c: Ag nanoparticle-decorated three-dimensional mesoporous silica gel.
d: A single- or multicore structures via coating Au with Zeolitic imidazolate framework (ZIF).
e: Ultrathin SnO_2-wrapped Au nanoparticles film.
f: Au nanoparticle-doped 3D-CMA semiconducting SnO_2 nanowire frameworks.
g: The bifunctional copper hydroxide/gold ordered nanoarray composites (this work).

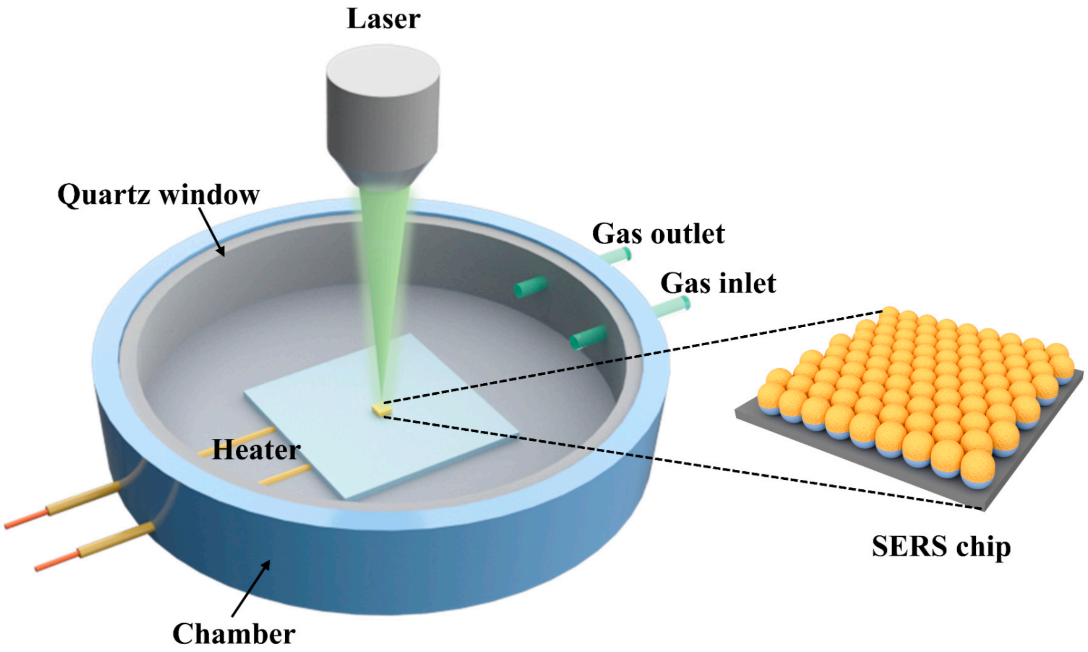

Scheme S1. Schematic illustration of the setup for the in-situ Raman spectral measurements of benzene-related VOCs.

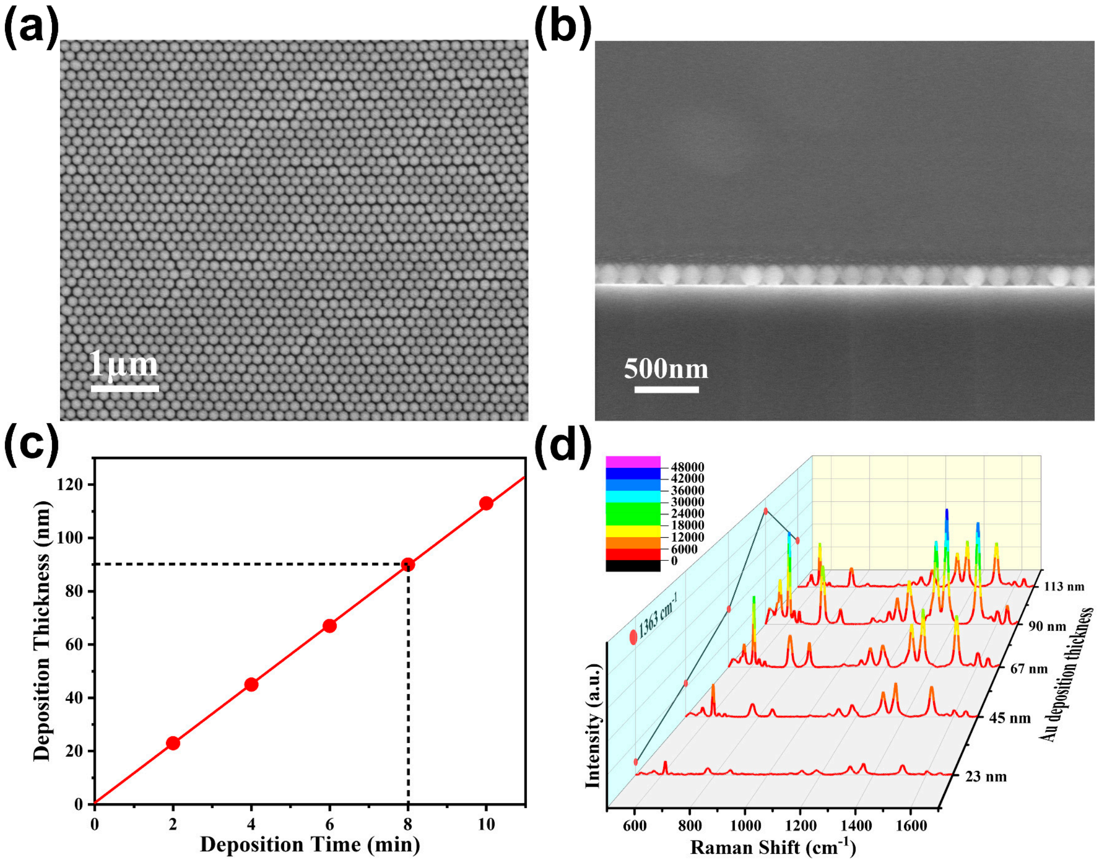

Figure S1. The FESEM image of (a) the SiO_2 colloidal monolayer on a Si wafer. (b) Cross-section view of the SiO_2 colloidal monolayer. (c) The linear plot of the Au deposition thickness versus deposition time. (d) The Raman spectra of the R6G-soaked Au/SiO_2 with different Au deposition thicknesses. The inset of (d) is the peak intensity at 1363 cm^{-1} versus Au deposition thickness.

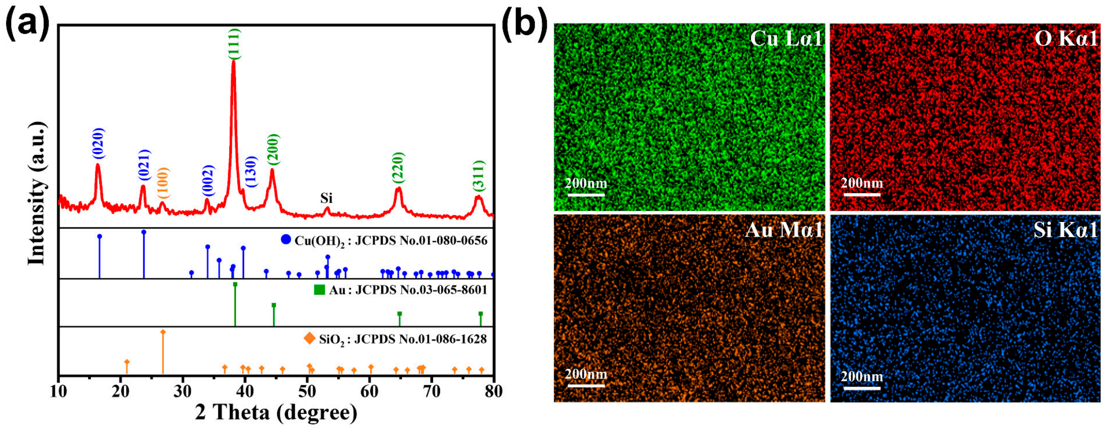

Figure S2. XRD pattern (a) and EDS elemental mapping demonstrations (b) of the Copper Hydroxide/Gold Ordered Nanoarray Composites.

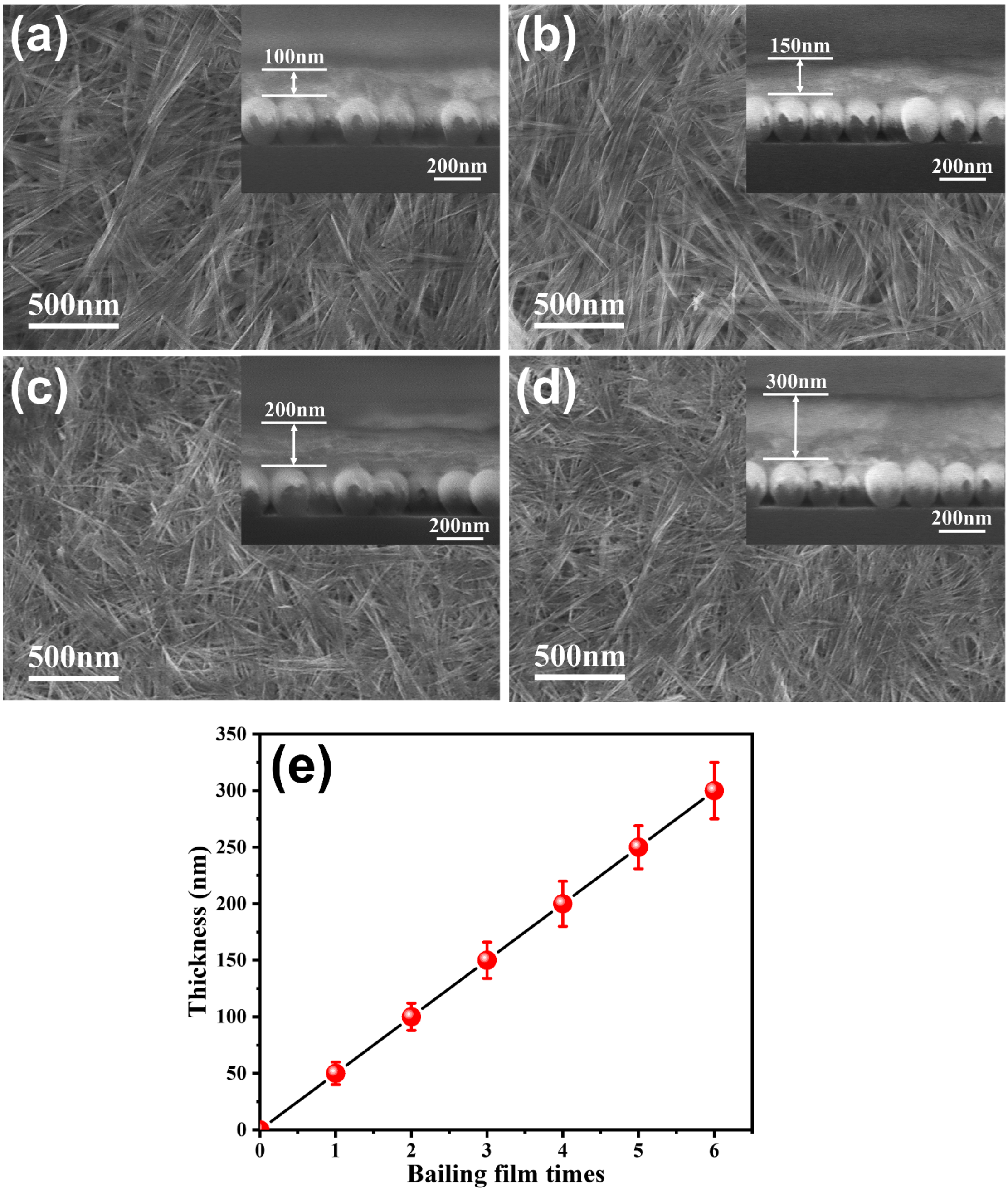

Figure S3. The covering-thickness control of the $Cu(OH)_2$-covered Au/SiO_2. (a-d) The FESEM images of the $Cu(OH)_2$-covered Au/SiO_2 with different thicknesses of $Cu(OH)_2$ covering layers. The insets are the corresponding cross-sectional views. (e) The $Cu(OH)_2$ covering thickness versus the bailing film times.

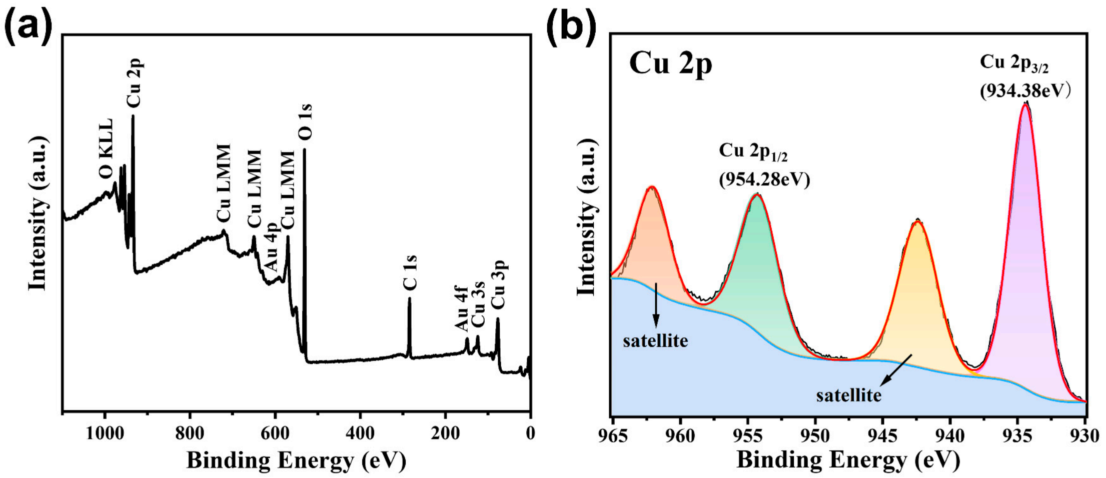

Figure S4. (a) The XPS measurement of the $Cu(OH)_2$-covered Au/SiO_2. (b) Binding energy spectra of Cu 2p.

Reference

[S1] Oliveira, A. P. S.; Gomes, I. S.; Neto, A. S. B.; Oliveira, A. C.; Filho, J. M.; Saraiva, G. D.; Soares, J. M.; Tehuacanero-Cuapa, S., Catalytic performance of MnFeSi composite in selective oxidation of styrene, ethylbenzene and benzyl alcohol. Mol. Catal. 2017, 436, 29-42.

[S2] Weber, V.; Brigo, L.; Brusatin, G.; Mattei, G.; Pedron, D.; Pilot, R.; Signorini, R., Hybrid Sol-Gel Surface-Enhanced Raman Sensor for Xylene Detection in Solution. Sensors 2021, 21 (23), 7912.

[S3] Gao, P.; Gosztola, D.; Weaver, M. J., Surface-Enhanced Raman-Spectroscopy as a Probe of Electro-organic Reaction Pathways. 1. Processes Involving Adsorbed Nitrobenzene, Azobenzene, and Related Species. J. Phys. Chem. C 1988, 92 (25), 7122-7130.

[S4] Zhang, M. F.; Liu, Y. K.; Jia, P. D.; Feng, Y. C.; Fu, S.; Yang, J.; Xiong, L. Z.; Su, F. M.; Wu, Y. C.; Huang, Y. J., Ag Nanoparticle-Decorated Mesoporous Silica as a Dual-Mode Raman Sensing Platform for Detection of Volatile Organic Compounds. ACS Appl. Nano Mater. 2021, 4 (2), 1019-1028.

[S5] Kim Y Y, Kim M K, Shin H S. Determination of Volatile Organic Compounds (VOCs) Levels from Various Smoking Cessation Aids by Using Gas Chromatography-mass Spectrometry Methodology. J. Toxicol. Environ. Health Part A 2022, 85, 110-120.

[S6] Lei, B.; Zhang, H.; Zhao, Q.; Liu, W.; Wei, Y.; Lu, Y.; Xiao, T.; Kong, J.; Cai, W., Facile Synthesis of ZnO/WO_3 Nanocomposite Porous Films for High-Performance Gas Sensing of Multiple VOCs. Nanomaterials (Basel, Switzerland) 2023, 13 (4), 733.

[S7] Rao, Z.; Liu, L.; Xie, J.; Zeng, Y. Y. Development of a Benzene Vapour Sensor Utilizing Chemiluminescence on Y_2O_3. Luminescence. 2008, 23, 163-168.

[S8] Zhou, Y.; Gu, Q. Y.; Qiu, T. Z.; He, X.; Chen, J. Q.; Qi, R. J.; Huang, R.; Zheng, T. T.; Tian, Y., Ultrasensitive Sensing of Volatile Organic Compounds Using a Cu-Doped SnO_2-NiO p-n Heterostructure That Shows Significant Raman Enhancement. Angew. Chem. Int. Ed. 2021, 60 (50), 26260-26267.

[S9] Chen, Q. Q.; Hou, R. N.; Zhu, Y. Z.; Wang, X. T.; Zhang, H.; Zhang, Y. J.; Zhang, L.; Tian, Z. Q.; Li, J. F., Au@ZIF-8 Core-Shell Nanoparticles as a SERS Substrate for Volatile Organic Compound Gas Detection. Anal. Chem. 2021, 93 (19), 7188-7195.

[S10] Bao, H.; Zhang, H.; Zhang, P.; Fu, H.; Zhou, L.; Li, Y.; Cai, W. Conductometric Response-Triggered Surface-Enhanced Raman Spectroscopy for Accurate Gas Recognition and Monitoring Based on Oxide-wrapped Metal Nanoparticles. ACS Sens. 2020, 5, 1641-1649.

[S11] Han, H. J.; Cho, S. H.; Han, S.; Jiang, J.; Lee, G.R.; Cho, E.N.; Kim, S.; Kim, D.; Jang, M.S.; Tuller, H. L.; Cha, J. J.; Jung, Y. S.

Synergistic Integration of Chemo-Resistive and SERS Sensing for Label-Free Multiplex Gas Detection. Adv. Mater. 2021, 33, 2105199.
